# Detection of biological signals from a live mammalian muscle using an early stage diamond quantum sensor

**DOI:** 10.1038/s41598-021-81828-x

**Published:** 2021-01-28

**Authors:** James Luke Webb, Luca Troise, Nikolaj Winther Hansen, Christoffer Olsson, Adam M. Wojciechowski, Jocelyn Achard, Ovidiu Brinza, Robert Staacke, Michael Kieschnick, Jan Meijer, Axel Thielscher, Jean-François Perrier, Kirstine Berg-Sørensen, Alexander Huck, Ulrik Lund Andersen

**Affiliations:** 1 Center for Macroscopic Quantum States (bigQ), Department of Physics, Technical University of Denmark, Kgs. Lyngby, Denmark; 2 Department of Neuroscience, University of Copenhagen, Copenhagen, Denmark; 3 Department of Health Technology, Technical University of Denmark, Kgs. Lyngby, Denmark; 4 Jagiellonian University, Krakow, Poland; 5 Laboratoire des Sciences des Procédés et des Matériaux, Université Sorbonne Paris Nord, 93430 Villetaneuse, France; 6 Division Applied Quantum System, Felix Bloch Institute for Solid State Physics, Leipzig University, 04103 Leipzig, Germany; 7 Danish Research Centre for Magnetic Resonance, Centre for Functional and Diagnostic Imaging and Research, Copenhagen University Hospital Hvidovre, Copenhagen, Denmark

**Keywords:** Biophysics, Optics and photonics, Physics

## Abstract

The ability to perform noninvasive and non-contact measurements of electric signals produced by action potentials is essential in biomedicine. A key method to do this is to remotely sense signals by the magnetic field they induce. Existing methods for magnetic field sensing of mammalian tissue, used in techniques such as magnetoencephalography of the brain, require cryogenically cooled superconducting detectors. These have many disadvantages in terms of high cost, flexibility and limited portability as well as poor spatial and temporal resolution. In this work we demonstrate an alternative technique for detecting magnetic fields generated by the current from action potentials in living tissue using nitrogen vacancy centres in diamond. With 50 pT/$\sqrt{\text {Hz}}$ sensitivity, we show the first measurements of magnetic sensing from mammalian tissue with a diamond sensor using mouse muscle optogenetically activated with blue light. We show these proof of principle measurements can be performed in an ordinary, unshielded lab environment and that the signal can be easily recovered by digital signal processing techniques. Although as yet uncompetitive with probe electrophysiology in terms of sensitivity, we demonstrate the feasibility of sensing action potentials via magnetic field in mammals using a diamond quantum sensor, as a step towards microscopic imaging of electrical activity in a biological sample using nitrogen vacancy centres in diamond.

## Introduction

Sensing of signals produced by living tissue is an essential tool for both medical diagnostics and for advancing the fundamental understanding of the structure and functioning of biological systems. Such signals, generated by propagating action potentials, are of particular importance in excitable cells such as neurons and muscle cells, allowing the cell-to-cell communication and movement that is essential for the functioning of the tissue and the organism as a whole^1^. Action potential can be measured to high precision and sensitivity using electrical probes, including by non-contact methods in proximity to neural pathways^2^. However, electrical probes are difficult to use for measurements of activity within tissue. Here the electrodes must pass through the tissue and any protective layer (e.g. the bone of the skull for a living subject), with a high risk of inducing damage. In the case of dissected samples for microscopy, insertion of electrodes can produce erroneous results or kill neurons near the probe^3^. For living subjects, this can have a severe impact on health, arising either from direct damage or secondary infection, particularly those arising from breaching the blood-brain barrier^4^. A further disadvantage is limited spatial resolution offered by a single point or multielectrode arrays^5^. Correctly positioning thin, flexible probes to reach a precise measurement site inside tissue is very difficult, with multielectrode arrays further adding to the invasiveness of the technique.

Magnetic field sensing provides an alternative route towards noninvasive, high resolution, high speed sensing. Here the magnetic field from electrical activity in any part of the tissue, including deep within it, can be detected to high precision and spatially localised without contact, from outside the specimen or subject and without the need to insert invasive probes. To date, techniques for sensing biological magnetic fields, for example magnetoencephalography (MEG) of the living brain, have been primarily based on superconducting quantum interference devices (SQUIDs)^6–9^.This approach requires bulky magnetic shielding and cryogenic cooling, thus preventing proximity studies of living tissue and delivering poor spatial resolution. These disadvantages have also limited the use of the technique for dissected tissue microscopy.

Noninvasive detection of magnetic fields in an unshielded, ambient environment can instead be realized by using nitrogen vacancy (NV) centres in diamond for magnetic field sensing^10–12^. NV centers are quantum defects that can provide broadband vector magnetic field sensing^13–16^ and imaging with high spatial resolution under ambient conditions using the technique of optically detected magnetic resonance (ODMR)^17,18^. It has broad applicability in life science^19,20^ particularly due to the high biocompatibility of diamond, which can be placed in contact or even within biological specimens^21,22^.Thus far NV sensing has focused on static or slow processes, such as imaging magnetotactic bacteria^23,24^. As yet there has been limited demonstration of sensing biological electrophysiological signals via magnetic field using diamond, with the most notable work being that by Barry et al.^25^ for invertebrates. Difficulties have included reaching sufficient sensitivity, keeping the sample alive and undamaged during measurement, interference from stimulation artifacts and the presence of background magnetic noise.

In this work, we report the first use of a diamond quantum sensor to measure action potentials *in vitro* from a live mammalian specimen via their magnetic field. We detect the induced field from the dissected leg muscle of a genetically modified mouse, using optogenetic stimulation of channelrhodopsin to induce the action potential through blue light stimulation. We achieve a magnetic field sensitivity of 50 pT/$\sqrt{\text {Hz}}$ and demonstrate methods that allow the specimen to remain alive under continuous measurement by the sensor. By using advanced data post-processing and filtering, we are able to demonstrate the first example of sensing of magnetic field from optogenetic stimulation using such a sensor under ambient conditions in a noisy, unshielded laboratory. Although as yet uncompetitive with probe electrophysiology in terms of sensitivity at this early stage, we consider these measurements an early but important step towards the goal of *in vivo* biosensing from living specimens using diamond quantum sensing, with the particular end goal of demonstrating sensing and imaging of signals in neural networks in the mammalian brain^26,27^.

**Figure 1:**
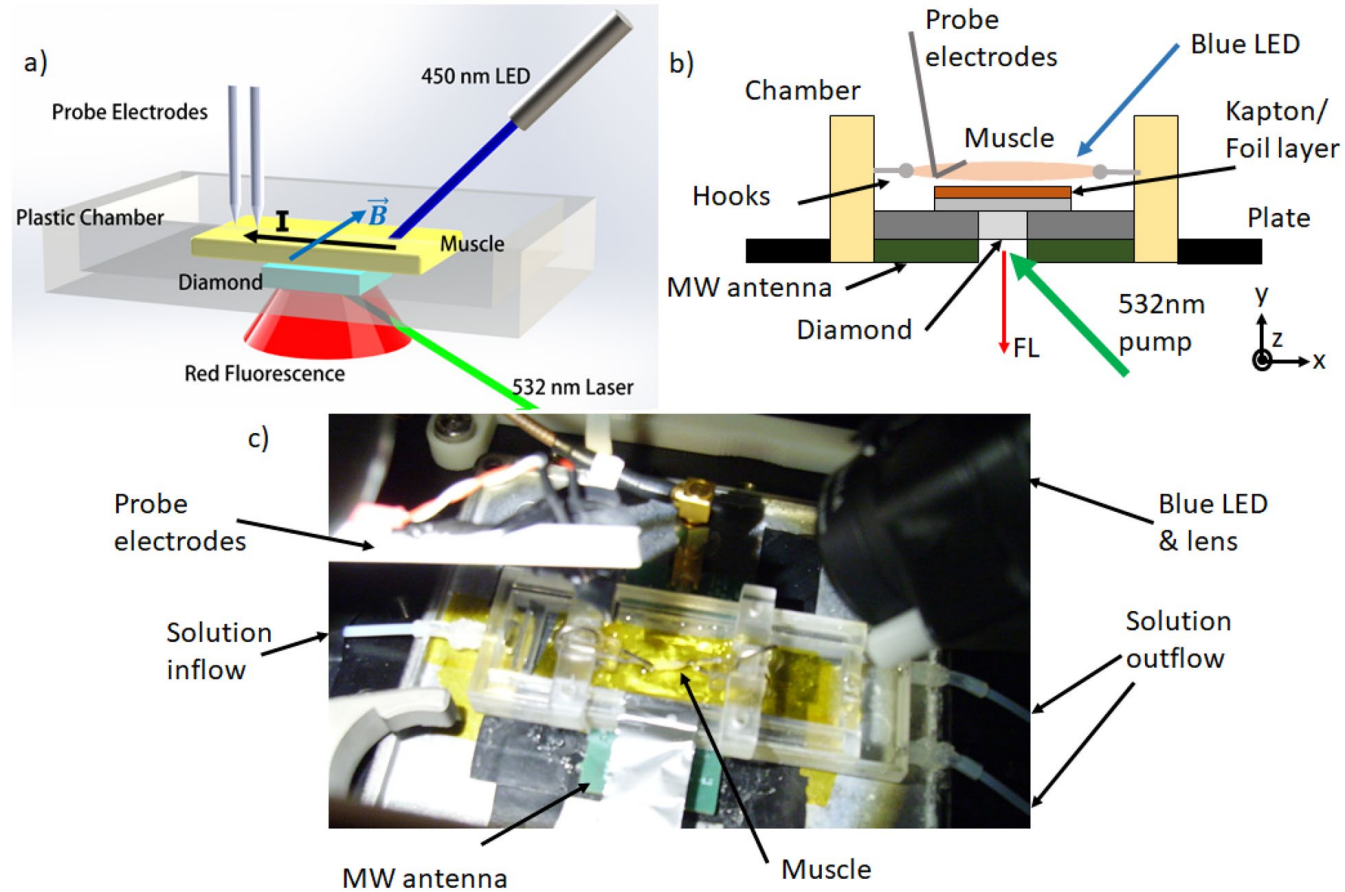
Experiment schematic and photograph. (**a**) Simplified 3D schematic of the magnetometer setup, showing the laser and blue LED illumination and fluorescence (FL) collection directions and sample chamber orientation, the direction of maximum magnetic field sensivity (B) and the direction of current flow (I) in the muscle. (**b**) Side view schematic (not to scale) of the chamber/diamond sensor/MW antenna stack, joined and affixed to a movable plate with silicone. (**c**) Photograph from above of the chamber, showing solution inflow connections and the mouse muscle, below which the diamond lies separated by a layer of Kapton tape and aluminium foil acting as a heatsink.

**Figure 2:**
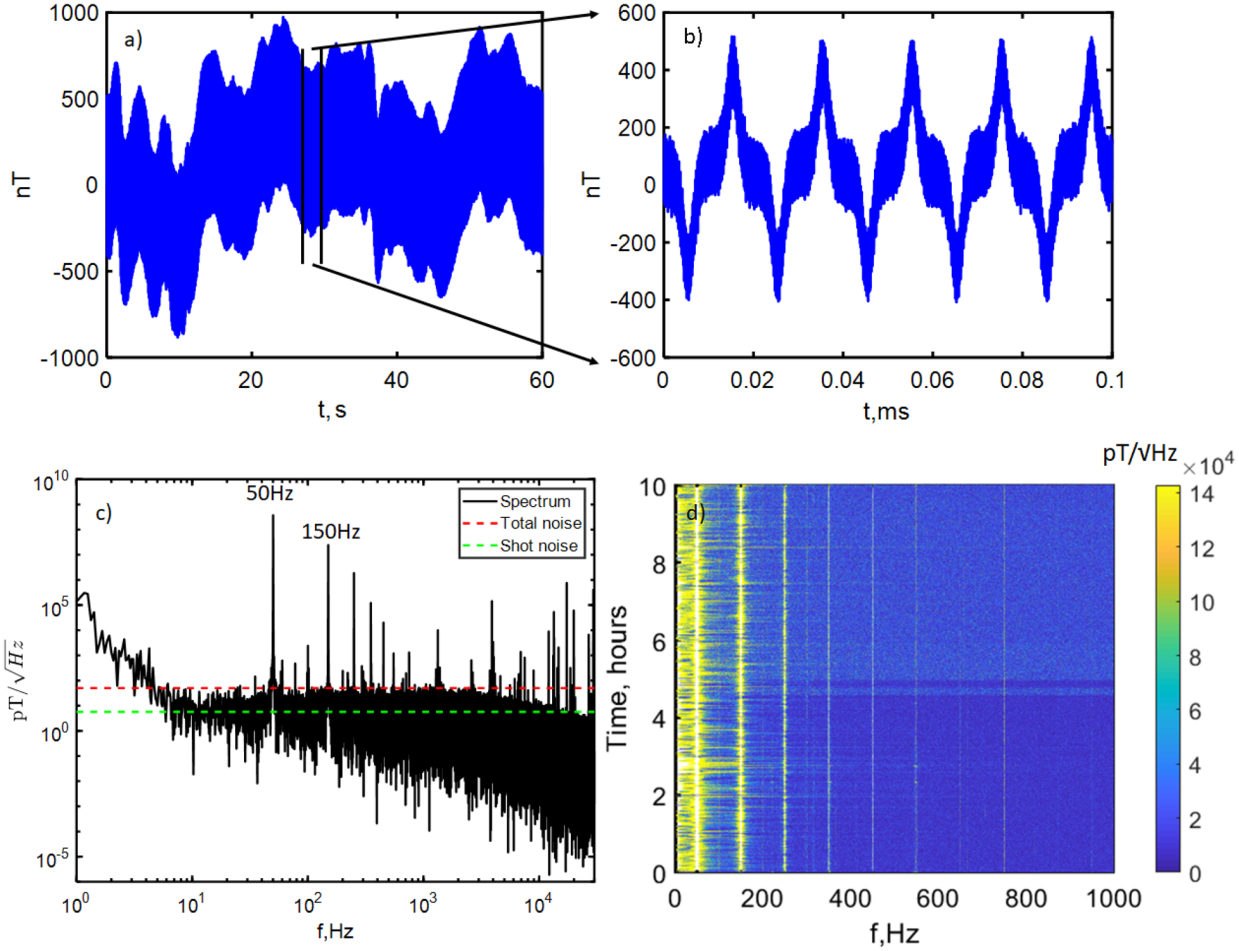
Demonstration of high dynamic range, bandwidth and sensitivity to magnetic field. (**a**) The raw unfiltered magnetic signal for a full 60 s iteration and (**b**) a zoomed 0.1 s segment of the same iteration. The signal was dominated by low frequency and DC laser power drift ($<5$ Hz), 50 Hz and 150 Hz noise from mains electricity and higher frequency ($>10$ kHz) noise and ranges between $\pm 1\,\upmu \hbox {T}$, well within the dynamic range of the magnetometer. (**c**) Spectral density in pT/$\sqrt{\text {Hz}}$ for a single 60 s iteration and (**d**) a spectrogram of repeated 60 s acquisitions over 10 h. The sensitivity floor is approximately 50 pT/$\sqrt{\text {Hz}}$ with f($-\,3\,\text {dB})=4.8$ Hz defined by the lock-in amplifier low pass filter. Also indicated are calculations of the total noise, which includes electronic and shot noise, and of the estimated shot noise level alone. Many sources of background magnetic noise can be seen to peak well above this floor.

## Results

We used an inverted microscope containing a diamond magnetic field sensor, consisting of a single crystalline diamond sample with a $20\,\upmu \hbox {m}$ layer comprising a high density of NV centers at the top facet (Fig. 1, see “Methods” section). The biological specimen was placed near the NV surface separated only by a foil/insulator layer, thereby ensuring high proximity of the specimen to the sensing NV layer. Laser light at 532 nm and frequency swept microwaves were applied to the NV sensor, while the induced fluorescence from the NV centers was imaged onto a photodetector. The time-varying magnetic field from the specimen was then detected using the protocol of optically detected magnetic resonance (ODMR) magnetometry.

### Dynamic range and background noise

The diamond sensor was capable of measuring all the ambient background noise up to the kHz frequency range while maintaining maximum sensitivity without the sensor signal output saturating. An example of the raw magnetometer signal measured can be seen in Fig. 2a,b. Assuming an ODMR linewidth of 1 MHz, the approximate dynamic range without loss of sensitivity was estimated as $42\,\upmu \hbox {T}$, comfortably above the 600 nT level of the predominant 50 Hz and 150 Hz background noise.

We first measured the background noise detected by the magnetometer with deionised water in the chamber but without a muscle. Figure 2c shows the amplitude spectral density, measured at microwave frequency on resonance (magnetically sensitive). Our noise floor was approximately 50 pT/$\sqrt{\text {Hz}}$, defined by contributions from the electronic noise of the amplifiers and photodetector and the shot noise of the detected fluorescence. Here the shot noise limited sensitivity was approximately 8 pT/$\sqrt{\text {Hz}}$. To characterise the noise, we measured the magnetometer output over many hours. The result can be seen in the spectrogram in Fig. 2d, showing the range ($<1$ kHz) where we expected to observe a biological signal. The two largest noise peaks can be seen at 50 Hz and 150 Hz, as expected from magnetic field detection of the fundamental mains frequency and from field produced by equipment transformers, each peak broadened by variable phase drift. Aside from mains harmonics, we observe a number of broad and narrowband noise sources. The majority of these we attribute to variable load airconditioning and water pumps in the building where the experiment was located, including some equipment from United States manufacturers that produced 60 Hz fields. We consider this background typical for a research lab or clinical environment.

**Figure 3:**
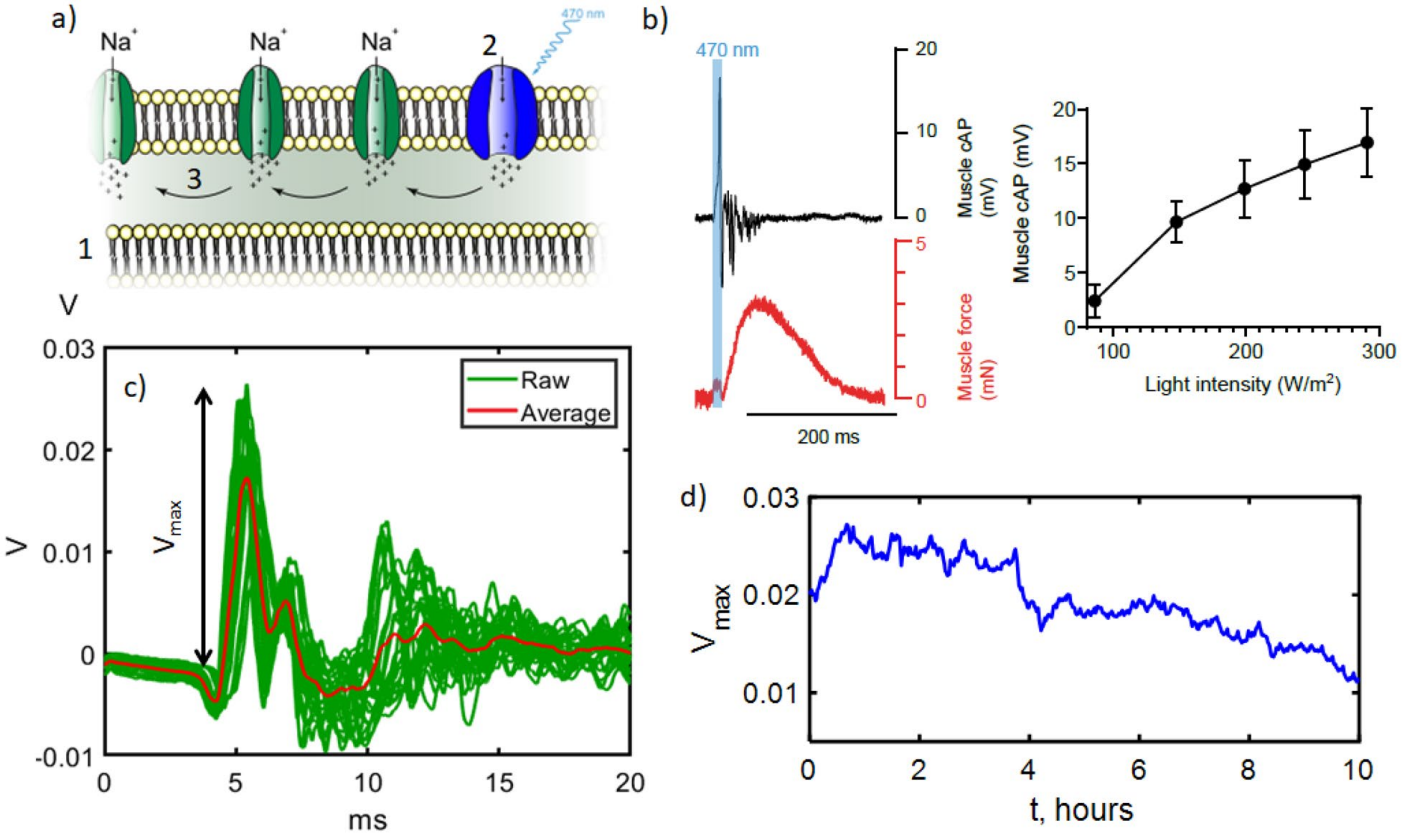
Mouse muscle electrophysiology and signal variation over time (**a**) Sketch of the biological signal generation process. In the muscle cell bi-lipid membrane (1) channelrhodopsin (2) opening triggers influx of $\hbox {Na}^{+}$ ions (3), creating an action potential running along the muscle. (**b**) Preliminary measurements taken on a separate setup of a single stimulation and readout via electrical probes (mV) and via muscle contraction force (mN). The strength of the signal as a function of light intensity is also shown. (**c**) Example of the readout of the biological signal in the magnetometer setup from a muscle (Muscle 1) by the electrical contact probe. Here $\hbox {t}=0$ ms is when the stimulation light is applied. The red trace shows the average signal observed over all stimulations. (**d**) (left axis) Maximum size of the initial peak in the signal, which steadily drops by a factor of 2 over time as the muscle becomes fatigued.

**Figure 4:**
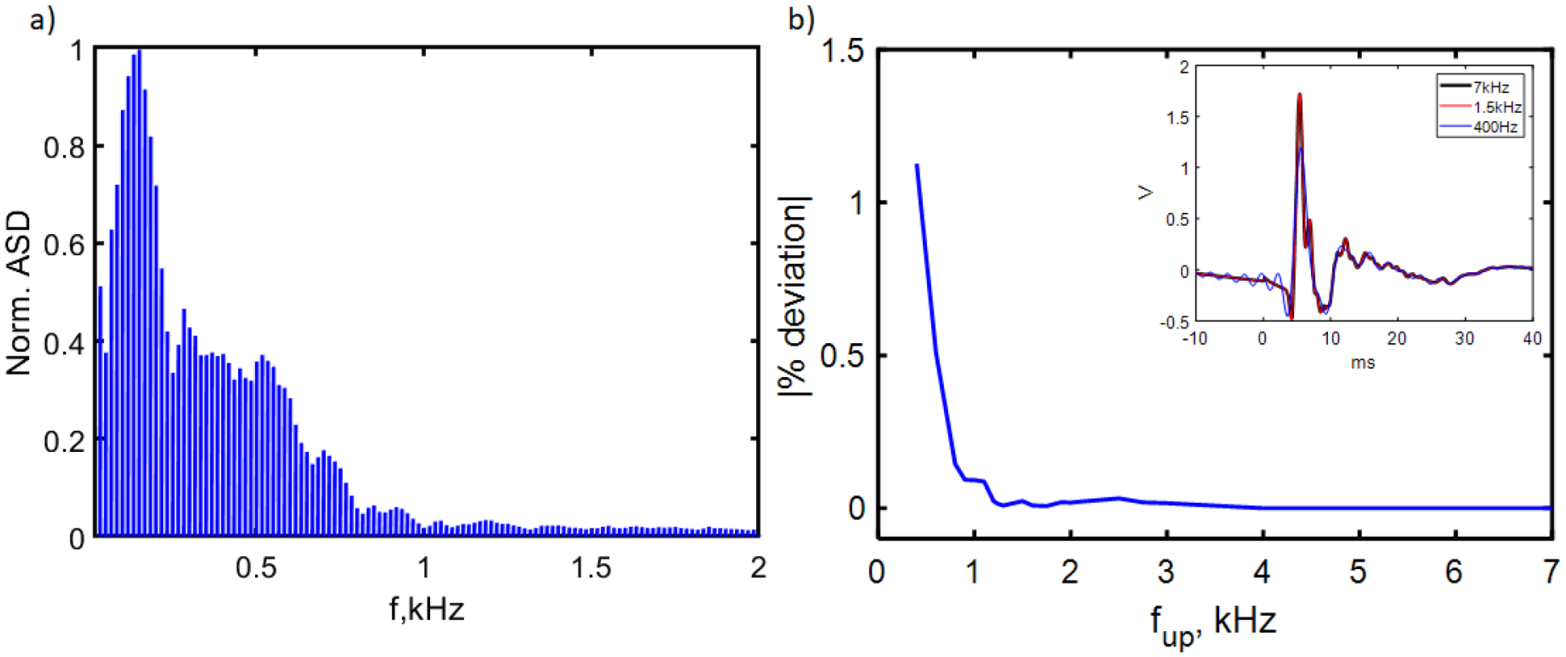
Frequency spectrum of the biological signal and defining optimum filter thresholding. (**a**) Spectrogram of the normalised Fourier transform amplitudes of the electrical probe voltage data, showing that the majority of the signal frequency components (shaded blue region) are under 1.5 kHz. (**b**) Percentage deviation from the unfiltered signal as a function of upper bandpass cutoff frequency $\hbox {f}_{{up}}$. The signal begins to be significantly corrupted below 1.5 kHz, as can be seen in the inset example for $\hbox {f}_{{up}}=400$ Hz where $\hbox {t}=0$ is the stimulation time.

**Figure 5:**
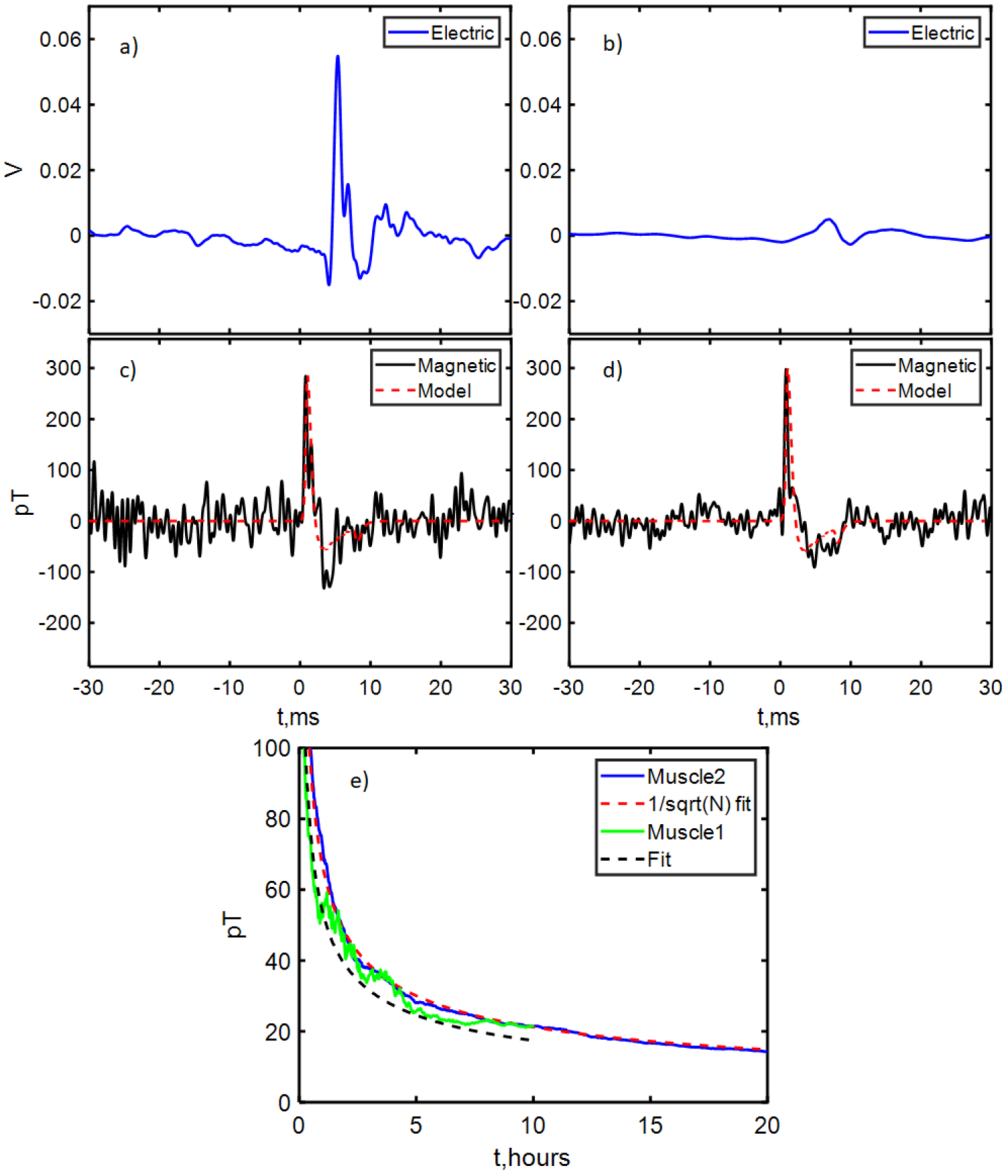
Simultaneous electrical and magnetometer readout of the biological signal. Here the blue traces in (**a**,**b**) are the scaled electrical probe data, with with black traces in (**b**,**c**) the simultaneous magnetometer readout for LED stimulation of two muscles: Muscle 1 (**a**,**c** left panes), averaged for 8 h ($30 \times 425$ stimulations) and Muscle 2 (**b**,**d** right panes), averaged for 16 h ($30\times 837$ stimulations). The maximum signal strength was approximately 250 pT. (**e**) Noise on the filtered magnetic data as a function of time for Muscle 1 and Muscle 2, showing 1/$\sqrt{N}$ dependence with the number of measurements taken (increasing time).

### Muscle electrophysiology

Figure 3a shows a sketch of the fundamental biological process to be measured, where stimulation with light triggers a cascading opening of ion channels, generating an action potential (producing current flow and magnetic field) along the muscle. Further details on this process are given in Supplementary Information. Prior to the magnetometry experiment, this optogenetic stimulation was tested in a preliminary investigation in a separate setup capable of measuring action potential and muscle extension force. An example of a stimulation, measuring action potential using electrical probes and by measuring the force resulting from subsequent muscle contraction can be seen in Fig. 3b . This test setup was used to determine the intensity of light required for good stimulation. No saturation in the electrical probe signal was observed up to the maximum intensity the light source could deliver.

Figure 3c shows the response measured using an electrical probe contact to a stimulated muscle in the magnetometer sample chamber. We measured both the diamond sensor output and the electrical probe contact simultaneously, to give a complete picture of the muscle behaviour. The maximum biological signal amplitude as measured by the electrical probes versus time is given in Fig. 3d. The signal strength decreased over time as the muscle fatigued. This meant that after a certain time, a maximum signal to noise ratio was reached where further averaging would not help resolve the biological signal in the magnetic data. To find this point, we calculated the signal to noise ratio of the signal as a function of number of iterations during postprocessing. The rate of fatigue varied between different muscles, ranging from 8 h in Fig. 3 up to 16–18 h. We note that our electrical probe data was taken purely as a reference to compare to the magnetic readout. It was measured via a simple DC measurement using a home-built amplifier, making it far noisier than specially designed RF shielded, state of the art electrophysiology setups.

### Filtering process

Figure 4a shows the amplitude spectral density from Fast Fourier transforming the electrical probe data. The majority of the signal can be found in a frequency range from DC up to hundreds of Hz (blue histogram plot), thus unfortunately overlapping with the majority of the background magnetic noise. We make the reasonable assumption that the magnetic readout resembles the electrical probe readout since they originate from the same biological process. Therefore to filter the magnetic data, we limited the bandwidth to the range in which we expect a signal, thereby rejecting the majority of the background noise. Postprocessing the data collected, we imposed a digital bandpass filter from $\hbox {f}_{{low}}=20$ Hz to a range of upper cutoff frequencies to determine the minimum at which the filter begins to corrupt the electrical probe data. This can be seen in Fig. 4b in the inset and as a percentage deviation from the raw signal in the main figure. We chose an upper cutoff of $\hbox {f}_{{up}}=1.5$ kHz, to include as many of the signal frequencies as possible. It can be seen clearly from the spectrum in Fig. 4a that this was more than sufficient to resolve the signal while excluding a significant amount of background noise.

In order to remove the background noise within the measurement bandwidth, we Fourier transformed each 60 s iteration dataset, selectively applied frequency domain notch filters corresponding to the noise peaks and then inverse Fourier transformed the data to recover the a filtered version of the timeseries. Due to the frequency overlap between signal and noise, it was critical to remove only parts of the signal that met two strict conditions: (1) to be clearly defined as noise (peak sufficiently above the white noise floor) and (2) only at those frequencies that did not distort the sought biological signal (on applying the same filter to the electrical probe data). Meeting only condition 1 would minimise noise while also removing the sought biological signal, whereas meeting only condition 2 would artificially recover the biological signal in the magnetic data by selection.

We met these conditions by using two threshold values. The first, $\hbox {n}_{{th}}$ we define as the multiple above the median spectral amplitude a peak must exceed to be classed as noise. To apply this, we divided the spectrum into 40 Hz wide windows, taking the median in each window $\hbox {m}_v$ and removed only those frequencies in each window that peaked above $\hbox {m}_{{v}}\times \hbox {n}_{{th}}$. By windowing, we avoided an excessive biasing of the filtering towards lower frequencies, due to the background 1/$\hbox {f}^{x}$ spectral slope. The second threshold value, $\hbox {m}_{{th}}$, we define as the percentage change in electrical probe signal relative to the unfiltered signal over a 40 ms window which starts at the time of stimulation ($\hbox {t}=0$). These methods are clarified further in Supplementary Information.

We first removed those frequency components with the largest spectral amplitude (most likely to be noise) and continued until the SNR for each 60 s iteration was maximised, requiring between 60–200 notched frequencies. In the Supplementary Information, we show how this process can be simplified by first removing the broad 50/150 Hz mains noise through time domain filtering, flattening the spectrum in the $<200$ Hz range^28^. We note that excluding the noise at $<5$ Hz due to laser power fluctuations, 84 percent of the magnetic noise (4.8 kHz bandwidth) was confined to 50 Hz and 150 Hz harmonics. As an aside, we show in the Supplementary Information that it is possible to significantly reduce the noise and recover a signal through use of a (n $\times$ 50 Hz) comb of fixed-width notch filters. This configuration could be easily implemented in hardware for a sensor device for practical applications.

### Biological signal via magnetic field

The timeseries for $\hbox {N} \times 60$ s iterations for both electrical probe and magnetic data was then averaged. Data was obtained separately from two muscles. The results for Muscle 1 can be seen in Fig. 5a,c and for Muscle 2 in Fig. 5b,d. We observe 1/$\sqrt{N}$ scaling (Fig. 5e) reaching an ultimate (rms) noise level of 22 pT for Muscle 1 and 16 pT for Muscle 2. The improved sensitivity for Muscle 2 was obtained with 12 h more measurement averaging. For the second muscle, 2,3-Butanedione monoxime was added to the solution bath in order to inhibit movement without affecting the action potential. For Muscle 1, this compound was absent. A signal was observed in the magnetic data for both muscles typical of an action potential propagating along the muscle. This signal was present with and without muscle inhibitor, ruling out the signal being an artifact arising from muscle motion. For Muscle 2, a signal to noise ratio of 1 was reached after 32 iterations ($30 \times 32$ simulations, 36 min measurement time), defining SNR as the averaged signal strength divided by the standard deviation of the averaged background magnetic noise. We phenomenologically modeled the expected action potential magnetic signal, full details of which are given in the Supplementary Information. The model parameters were within the range provided by literature and yield good agreement to the experimental data^29–31^.

We note that for Muscle 1 the diamond was placed approximately 2mm±1 mm closer to the stimulation position along the muscle length than the electrical probes. This gap was not present for Muscle 2. The biological signal in the magnetic data for Muscle 1 was therefore consistently observed 1.5 ms±0.5 ms ahead of the electrical probe readout. This gives a crude estimate of propagation velocity in the muscle of 0.5–3 m/s^32^. The observed delay rules out that the recorded magnetic signal could stem from crosstalk pickup from the simultaneously measured electric probe circuit.

The difference in the shape and magnitude of the electrical readout between Muscle 1 and Muscle 2 arises from differences in contact quality between the muscles and the silver chloride probe electrodes. As a result of a reduced contact quality to Muscle 2, the signal strength was lowered and additional capacitance was introduced leading to distortions of the signal probed by the electrodes. This effect is not present on the magnetometer readout where we saw a sharp response, thus representing an advantage of the magnetic sensing over conventional electrophysiology.

## Discussion

Using a diamond quantum sensor with pT-scale sensitivity to magnetic field and kHz measurement bandwidth, this work provides the first demonstration of sensing of the magnetic field from a signal generated by a living, mammalian biological specimen. We show that the sample can be kept alive for many hours while being measured using the quantum sensor, despite the current need for high laser power. The signal resembles that typical of action potentials measured by conventional electrical probes, without the drawbacks of poor electrical contact adding capacitive distortion. We measure a time delay between magnetic and electrical probe signal consistent with signal propagation along the muscle. Using optogenetic activation and comparison to a muscle where motion had been inhibited ensured the signal we measure was free of artifacts. The magnetometry technique is not dependent on optogenetic stimulation and is widely applicable to conventional electrical probe stimulation, or where stimulation originates from the living specimen itself.

Using digital signal processing techniques, we show that a weak magnetic signal can be recovered in a noisy background without magnetic shielding, even in an ordinary laboratory environment with a significant degree of background magnetic noise typical of that in a large, busy building at a university or a hospital. Unlike alternative methods for high-sensitivity magnetometry, the high dynamic range of the diamond sensor allows the background noise to be recorded without saturation. Since the sensor does not saturate, the background noise can be directly detected and can thus be removed by adaptive windowed notch filtering. Future advances in sensitivity will only help improve the clear identification of the different sources of background noise, aiding filtering and noise reduction and could eventually allow active cancellation of magnetic noise in a small volume using a second sensor.

We obtain a peak-to-peak noise of between 200–300 pT on a single 60 s iteration after filtering. Such a noise level would allow a single stimulation event of 1–1.5 nT (with $\hbox {SNR}\ge 5$) to be clearly observed in realtime. As the majority of action potentials only drive sufficient current to produce a field in the sub-nanotesla range, some improvement is therefore required to reach single shot readout. However, if high proximity between the sample and sensor is achieved (as is possible in our setup) then nanotesla-level signals may be observed. Our previous theoretical calculations (Karadas et al.^27^) show signals of 1 nT from the hippocampus of mice and previous work using invasive probes close to neurons in living subjects has shown similar field strength^33^.

We show that filtering can also be done to a reasonable degree using fixed-width notch filters at mains harmonics frequencies. This could be implemented in hardware for realtime filtering in a portable sensor device to be used in a research or clinical environment. Using a higher quality, isotopically purified diamond would allow significant improvement in sensitivity over our previous work in this direction (see^34^). Although it may not be desirable or necessary to minaturise the setup for microscopy in order to maintain mechanical stability and ease of access for users, a portable handheld diamond sensor would allow recording of signals from living whole organisms, in particular for faster signals (kHz bandwidth and above) that cannot be easily detected by competing technology. As discussed in our previous work, feeding multiple diamond sensors from the same central laser via fibre optic coupling would be highly desirable, particularly in terms of spatially resolving the location of signals within tissue and to perform gradiometry to reduce common mode noise^35^. An alternative direction is full miniturisation and integration of the sensor using semiconductor nanofabrication techniques, although as yet this has yet to deliver the necessary sensitivity^36,37^.

The capability to operate in an ordinary lab or clinical environment without relying on superconducting technology, would open the door to many new research and diagnostic possibilities. A number of competing technologies seek to do this, most notably atomic vapour magnetometers^38–41^. Although they are thus far superior in sensitivity, compared to diamond NV sensing, they have a number of disadvantages such as lack of biocompatibility, low dynamic range, inability to perform vector sensing in a single sensor, the need to screen from the Earth’s magnetic field to achieve maximum sensitivity and low bandwidth at maximum sensitivity ($<100$ Hz for a recent commercial atomic vapour sensor from QuSpin, Inc.^42^ used for biosensing) that can be insufficient to achieve the micro to few-millisecond (kHz) time resolution needed to sense many biological signals.

We note that the measurements we present in this work could be easily performed with existing electrical probe electrophysiology techniques, to a far higher degree of sensitivity than via our magnetic measurements. However, it is not our intention for our method to be competitive with state of the art electrical probes, but to be a step towards offering new, noninvasive capabilities they cannot deliver, while having advantages over alternative magnetic sensing methods such as high bandwidth and room temperature operation. Our results represent an important proof of concept experiment, demonstrating that a diamond quantum sensor in a very early stage of development can sense mammalian biological signals. The end goal, for which this experiment represents an early step, is to use the diamond sensor to perform microscopic imaging of magnetic field, to give noninvasive high spatial resolution of electrical activity^26^. This would offer a new capability to map electrical activity, important for example in studying the structure in the brain and linking this with whole organism behaviour. Our setup is designed to eventually be capable of this imaging via widefield microscopy from dissected tissue slices. Alternative microscopy techniques are also being pursued towards this goal, in particular fluorescence microscopy from nanodiamonds within the biosample^22,43^ and non-contact scanning sensors^44^. Scanning sensor technology is currently limited to atomically flat surfaces, however advances in diamond probes, potentially arising from work on boron doped diamond probes used for invasive electrochemical sensing, may be of use in developing a non-invasive scanning NV bioprobe^45,46^. It is hoped that further advances in diamond materials development and through novel sensing schemes that a level of sensitivity can be reached to allow imaging using these methods in the coming years, with sufficient spatial resolution ($<10\,\upmu \hbox {m}$) to resolve signals along individual neural pathways^47^.

## Methods

### Inverted microscope

Figure 1a shows a simplified 3D schematic of our inverted microscope incorporating the diamond biosensor. For optical pumping, up to 2 W of horizontally polarised 532 nm green laser (Coherent Verdi G2) illumination could be delivered from below a raised platform at Brewster’s angle for diamond (67 deg). Polarisation was controlled before incidence on the diamond using a half wave plate to ensure maximum power transmission. Red fluorescence from the diamond was collected separately from the incident green light using an aspheric, anti-reflective coated 12 mm diameter condenser lens (Thorlabs ACL1210). Fluorescence light was directed onto an electronically balanced photodetector (New Focus Inc.). 6 mW was the typical power of collected fluorescence for 2 W of green laser light. A reference beam for the photodetector was obtained by splitting off a few-mW portion of the input beam using a polarising beamsplitter.

### Diamond preparation

The diamond used in this work was a [100] oriented electronic-grade single crystal from Element Six with dimensions $2 \times 2 \times$ 0.5 $\hbox {mm}^3$ overgrown by a $20\,\upmu \hbox {m}$ thick nitrogen doped layer using chemical vapor deposition (CVD). Nitrogen content in the gas phase was optimised during the growth to reach a level of 5ppm of nitrogen-14. The diamond was then 2.25 MeV proton irradiated with a fluence of $3 \times 10^{15}$ protons/$\hbox {cm}^{2}$ followed by annealing at 800 $^{\circ }$C for 4 h. This gave a $\hbox {NV}^{-}$ density ranging between 0.1 and 1 ppm. The diamond was mounted into a central hole of a laser cut aluminum nitrate heatsink plate of dimensions $3\times 3 \times 0.05\,\hbox {cm}^3$. We measured an ODMR linewidth of 1 MHz with a contrast of approximately 1.5 percent for each nitrogen-14 hyperfine transition.

### Sensor geometry

The diamond and aluminium nitride plate were attached using watertight aquarium-safe silicone to a custom built broadband microwave antenna fabricated onto a printed circuit board with a hole for fluorescence collection from below (see schematic Fig. 1b). On top of both antenna and plate was silicone mounted a rectangular 3D-printed, custom designed rectangular plastic sample chamber, which can be seen in Fig. 1c, that could hold a flow bath of solution, fed using a peristaltic pump. The chamber was fully accessible from above, allowing biological samples to be introduced and probe electrodes to contact the sample using micromanipulators. The sample was held on a pair of sliding hooks within the bath, directly above the top surface of the diamond. To protect the biological sample from laser heating, a $16\,\upmu \hbox {m}$ thick layer of aluminum foil was placed on the top surface of the diamond, attached by $50\,\upmu \hbox {m}$ Kapton tape in order to electrically insulate the foil and diamond from the sample. The resulting tens of micrometer separation between sample and diamond was undesirable due to reduction in magnetic field strength, but was taken as a precaution against sample heat damage based on previous experimental experience. We note that this layer is not waterproof, meaning the sample and diamond sensor are in indirect contact via the solution bath. Here the biocompatibility and robustness of the diamond sensor is an advantage, since it will not degrade or otherwise contaminate the solution which could ultimately kill or damage the biological sample to be studied. The layer could be reduced in thickness and ultimately eliminated by better conduction of heat away from the diamond and the use of lower laser power without compromising sensitivity via future diamond material development.

### Control and readout

The microwave field was generated using a three-frequency drive scheme^48^ using two radiofrequency (RF) generators (Stanford SG394) feeding a balanced mixer and then amplified (Minicircuits ZHL-16W-43+). One generator drove the transition between the $m_{s}=0$ and $m_{s}=\pm 1$ of the ground state of the NVs with a frequency in the range of 2.7–3 GHz and frequency modulated at 33 kHz to implement lock-in detection. The second generator provided a fixed frequency of 2.16 MHz to drive multiple hyperfine transitions. Two rare-earth magnets were aligned parallel to the (110) direction in the diamond and perpendicular to the main direction of signal current propagation, generating a DC bias field of $\sim$ 1.5 mT. These directions are labelled on Fig. 1a and the field axis corresponds to ther z-axis on Fig. 1b. We used a continuous wave (CW) scheme with constant microwave and laser power ensuring a stable (temperature) environment. Magnetometer sensitivity was optimised by adjusting the power of the reference beam to the balanced photodetector and by independently sweeping the power on the two RF signal generators to optimise microwave drive.

Finally, the output voltage from the balanced detector was passed to a lock-in amplifier (Stanford SR850), from which the output was digitised by an analogue to digital converter (ADC, model NI PCI-6221) at 80 kSa/s. We term this channel the *magnetic data*. We used a lock-in time constant of $30\,\upmu \hbox {s}$, giving a magnetic field measurement bandwidth of approximately 4.8 kHz. The muscle was surface contacted by an electrical probe consisting of two L-shaped AgCl coated silver wires positioned 3 mm apart under the muscle mounted on a micromanipulator. The recording electrode voltage was amplified (Axon Cyberamp 320). This was then digitised at the same rate and simultaneously with the magnetic data. We term this channel the *electrical probe data*.

### Specimen preparation

The muscle was stimulated optogenetically using blue light from a 470 nm LED. Experiments were performed on genetically modified mice in which Channelrhodopsin 2 (ChR2), a light-gated cation channel, was used to create an action potential in the muscle. Animals were euthanized by cervical dislocation and extensor digitorum longus (EDL) muscles from both hind limbs were dissected in carbogen-saturated (95$\%$ $\hbox {O}_{{2}}$/5$\%$ $\hbox {CO}_2$) cold artificial cerebrospinal fluid (ACSF). Small suture loops were tied on distal and proximal tendons for later suspension in the recording chamber. Until use, EDL muscles were stored in a holding chamber continuously bubbled with carbogen. For some muscles, the myosin ATPase inhibitor 2,3-Butanedione monoxime (5 mM in ACSF; Sigma) was added in order to uncouple excitation from contraction, ensuring that we measure only action potential and removing any possible artifacts arising from sample motion. Full details of the biological preparation are given in Supplementary Information with this work.

Prior to the experiment, the sample chamber and connecting tubing were cleaned by pumping heavily diluted household bleach through the system, followed by flushing with deionised water. This was then replaced with ACSF solution, carbogenated in a 500 ml bottle and forming a closed circuit with the sample chamber. Temperature was measured in the chamber as 34 $^{\circ }$C with laser and microwave power on. The mouse muscle was held in the chamber by suture loops on hooks just above (but not in contact with) the diamond.

### Stimulation protocol

The muscle was optically stimulated every 2 s, with a light pulse length of 5 ms. The absence of contamination of the recording by a photovoltaic effect (Becquerel effect) induced by light was confirmed by taking traces recorded with the same protocols in the absence of muscle. Data from the magnetometer and from the electrodes was recorded from the ADC for 60 s data acquisition iterations during stimulation, giving 30 stimulations per iteration. The full data from both magnetic and electrical probe channels ($2 \times 80 \hbox {kSa/s} \times 60$ s) was stored for postprocessing. Many hours of data aquisition was possible. Postprocessing ensured that unexpected transient noise could be captured. A random delay time was implemented between 60 s iterations (length between 10–30 s), ensuring each iteration began with a different mains phase to assist averaging. The absolute start time of each iteration was recorded and this measurement timeseries is used in the relevant plots in the results section. A fast ODMR sweep for selecting the optimal MW frequency was performed every 5 min during the measurement to compensate for any thermal drift.

### Ethical statement

All methods in this work were carried out in compliance with the ARRIVE guidelines according to relevant Danish national guidelines and regulations. Experimental protocols were approved where required by the Technical University of Denmark, the University of Copenhagen and the Danish National Committee on Health Research Ethics (DNVK).

## Supplementary Information

Supplementary Information 1.
