# Supplementary material for: Detection of biological signals from a live mammalian muscle using an early stage diamond quantum sensor
Source: Sci Rep. 2021 Jan 28;11:2412. doi: 10.1038/s41598-021-81828-x (PMC7844290; doi:10.1038/s41598-021-81828-x)
Supplement: Supplementary file 1 — Supplementary Information 1. [file 41598_2021_81828_MOESM1_ESM.pdf]

Supplementary Information: Detection of biological signals from a live mammalian muscle using an early stage diamond quantum sensor

James Luke Webb[1], Luca Troise[1], Nikolaj Winther Hansen[2], Christoffer Olsson[3], Adam M. Wojciechowski[4], Jocelyn Achard[5], Ovidiu Brinza[5], Robert Staacke[6], Michael Kieschnick[6], Jan Meijer[6], Axel Thielscher[3,7], Jean-François Perrier[2], Kirstine Berg-Sørensen[1], Alexander Huck[1], and Ulrik Lund Andersen[1]

[1]*Center for Macroscopic Quantum States (bigQ), Department of Physics, Technical University of Denmark, Kgs. Lyngby, Denmark*

[2]*Department of Neuroscience, University of Copenhagen, Copenhagen, Denmark*

[3]*Department of Health Technology, Technical University of Denmark, Kgs. Lyngby, Denmark*

[4]*Jagiellonian University, Krakow, Poland*

[5]*Laboratoire des Sciences des Procédés et des Matériaux, Université Sorbonne Paris Nord, 93430 Villetaneuse, France*

[6]*Division Applied Quantum System, Felix Bloch Institute for Solid State Physics, Leipzig University, 04103, Leipzig, Germany*

[7]*Danish Research Centre for Magnetic Resonance, Centre for Functional and Diagnostic Imaging and Research, Copenhagen University Hospital Hvidovre, Denmark*

1 Determination of filter threshold

We define a threshold value n_{th} as the multiple of the median of the PSD in each one of n=1500Hz/40Hz windows, starting at DC to the upper bandpass cutoff. This is illustrated in Supplementary Figure 1. In order to optimise the threshold values, we filtered the recorded data using different values of n_{th}. Averaging over all 60sec iterations, we calculated the maximum electrical probe signal strength, the percentage deviation from the unfiltered electrical probe signal (m_{th}) and the noise on the magnetic data, defined as the standard deviation in the timeseries in a 0.1ms period leading up to the stimulation. Plots of each of these values can be seen in Supplementary Figure 2,a),b) and d) as a function of n_{th} for Muscle 1. From the figures, it can be clearly seen from the data that for n_{th}<3, there is a considerable effect: although the magnetic noise drops, the electrical probe signal becomes weaker and distorted by the filtering imposed.

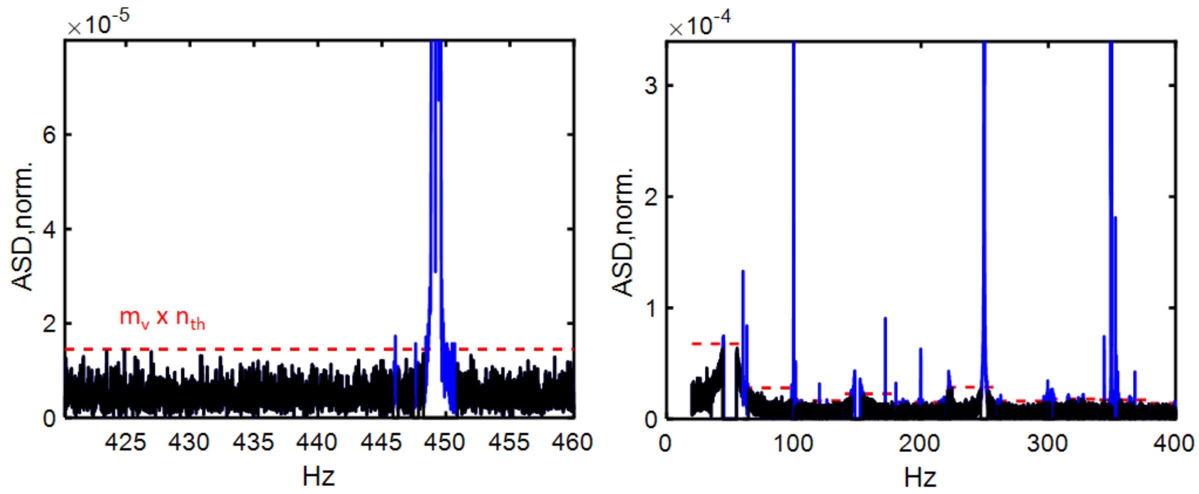

Supplementary Figure 1: Illustrated example of the window filtering procedure. A threshold (red, dashed) is defined as the median value m_v in a 40Hz window (here 420-460Hz) multiplied by a user-specified threshold value n_{th}. Any frequency components that exceed this line in the raw data (blue) are removed, giving the (black) filtered data. This removes strong noise peaks. In the left pane this is illustrated for the 9th mains harmonic at 450Hz. This is repeated in 40Hz steps starting at 20Hz. This procedure is exemplified in the right pane for f<400Hz. Here the 50Hz and 150Hz have been removed by a previous notch filter step. The threshold must be sufficiently low to eliminate noise peaks while not too low, which would remove the remaining signal. An advantage for our experiment is that the biological signal SNR for each iteration is <<1, meaning the threshold can be low and any peak significantly above the (white) noise floor can be removed.

We therefore choose n_{th}=3 for our filtering, which gives some improvement over the raw signal noise with minimal disruption to the signal, as exemplified when applied to the electrical probe data in Supplementary Figure 2,c). This value of n_{th} corresponds to a percentage deviation m_{th} of 0.8 percent. We found the filtering process to be relatively insensitive to window width, as long as the window was sufficient to include the full width of the 50Hz mains noise peak. Using 40Hz includes the majority of this peak in the second window. The method could likely be improved by using variable n_{th} for each window, at the cost of computation time.

2 Time domain filtering

As an optional initial step, we could remove the majority of the 50 and 150Hz mains background noise by matching and subtracting the signal in the time domain. This was done by monitoring the drift in phase of the mains signal during the experiment over each 60sec dataset by capacitively sampling the mains at a rate matching the capture of the magnetic and electrical probe data (80kSa/sec), then using this information to digitally generate a signal of matching phase and amplitude to the noise in the magnetic data that could then be subtracted from the magnetic data timeseries. For 150Hz it was necessary to repeat this process 3-5 times with slightly different constant phase delay added, due to this signal arising from multiple different transformers in

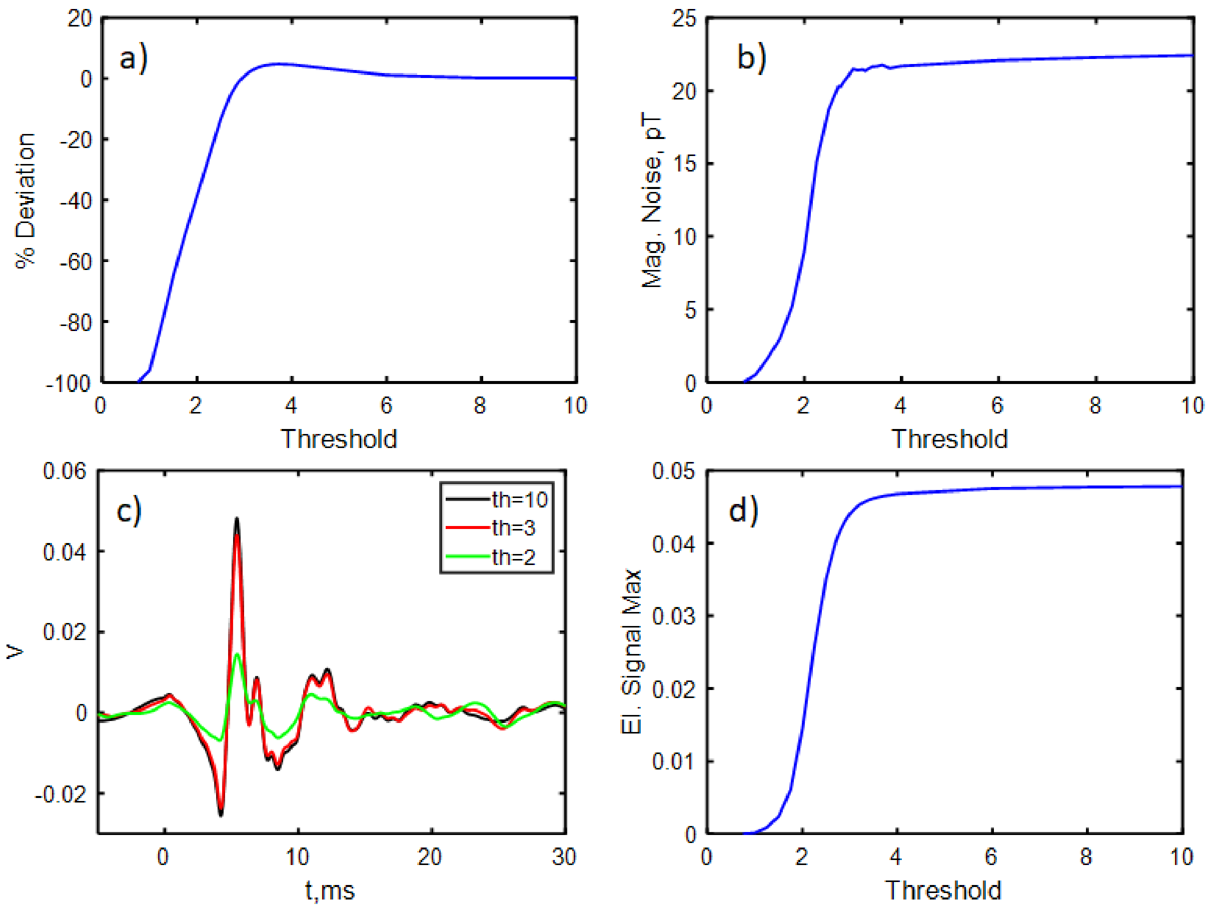

Supplementary Figure 2: Determination of filter threshold using data from Muscle 1. a) Percentage deviation m_{th} between the filtered and unfiltered electrical probe signal as a function of cutoff threshold (n_{th}). b) The filtered noise on the magnetic data as a function of n_{th}. c) Examples of the filtered electrical probe signal at different values of n_{th}. d) Maximum amplitude of the electrical probe signal as a function of n_{th}

equipment near to the magnetometer. The result of this process is exemplified in the spectrum in Supplementary Figure 3a) and b). This process greatly simplified subsequent notch filtering by removing the broad frequency spread of the 50 and 150Hz noise due to mains phase drift.

3 Mains notch filtering

A simpler method of filtering was to remove the noise only at mains harmonic frequencies using fixed width notch filters, since these carry the majority (84 percent for 50 and 150Hz) of the noise in the magnetic data. We implemented this scheme in postprocessing, by FFT of the data, zeroing the amplitudes of the target frequencies and inverse transforming. However, this is a scheme that could be easily implemented in realtime and in hardware, making it very suitable for a sensor device operating in a lab or clinical environment. The resulting signal can be seen in Supplementary Figure 4,c) for data from Muscle 1, with Supplementary Figure 4,d) the effect of applying the same filter to the electrical probe signal. To find the ideal filter, we

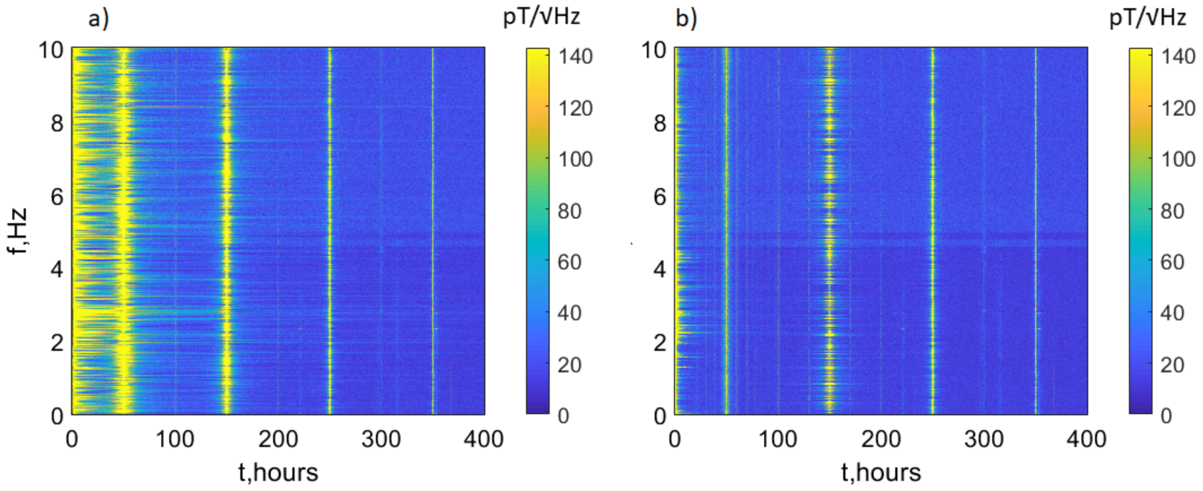

Supplementary Figure 3: Amplitude spectral density spectrogram of magnetic data at <400Hz a) before and b) after time domain filtering of the 50Hz and 150Hz signals using the mains phase recorded during the experiment. Although cancellation was not complete due to the difficulty of matching multiple out of phase sources, the noise was reduced and the spectrum flattened in the low frequency range.

varied the bandstop frequency width and number of frequencies in the comb and applied them to both magnetic and electrical probe data. The deviation observed by filtering the electrical probe signal can be seen in Supplementary Figure 4,a) and the variation in magnetic noise in 4,b). A minimum of 6 frequencies were required in the comb (50,100,150,200,250,300Hz) to observe the signal in the magnetic data, with the majority of the noise in the odd (transformer induction) harmonics . Although the noise was relatively insensitive to filter width >2Hz, as this covered the frequency range of the mains noise harmonics, the width needed to be minimised to avoid unnecessary distortion to the signal. Although we show the minimal case here, additional notch filters could be adaptively implemented to remove off-mains noise, such as from North American 60Hz mains or transient noise from air conditioning or pumps.

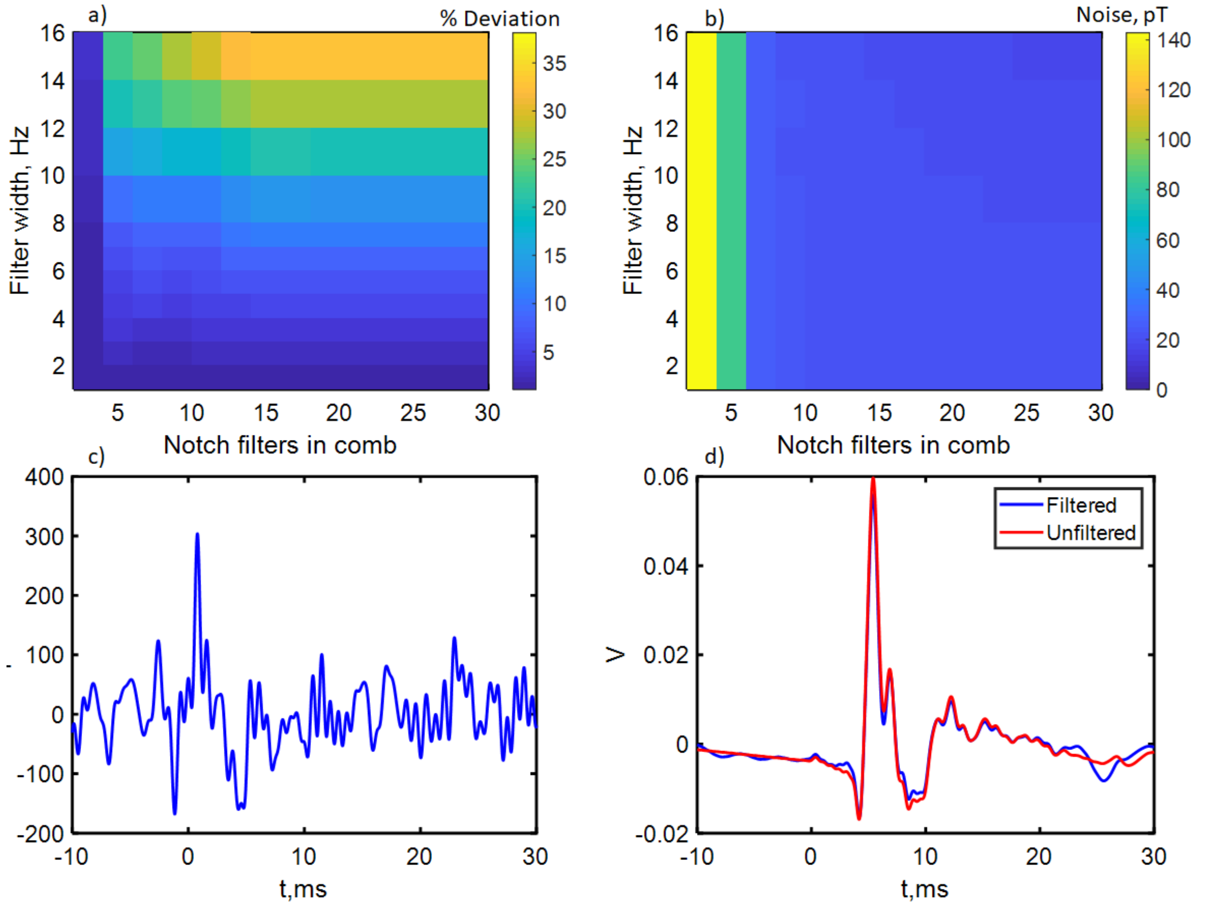

Supplementary Figure 4: Filtering by a comb of notch filters at mains harmonics frequencies for data from Muscle 1. a) Percentage deviation m_{th} from the unfiltered electrical signal and b) noise on the filtered magnetic data in pT as a function of number of notch filters in the comb and filter width in Hz. Noise was relatively insensitive to width >2Hz, which was enough to cover the noise peak width for the main harmonics. A minimum of 6 notches were required to reduce the noise to a level where the signal could be observed. This is shown in c), with the filtered electrical probe signal using the same filter shown in d) in comparison to the unfiltered signal.

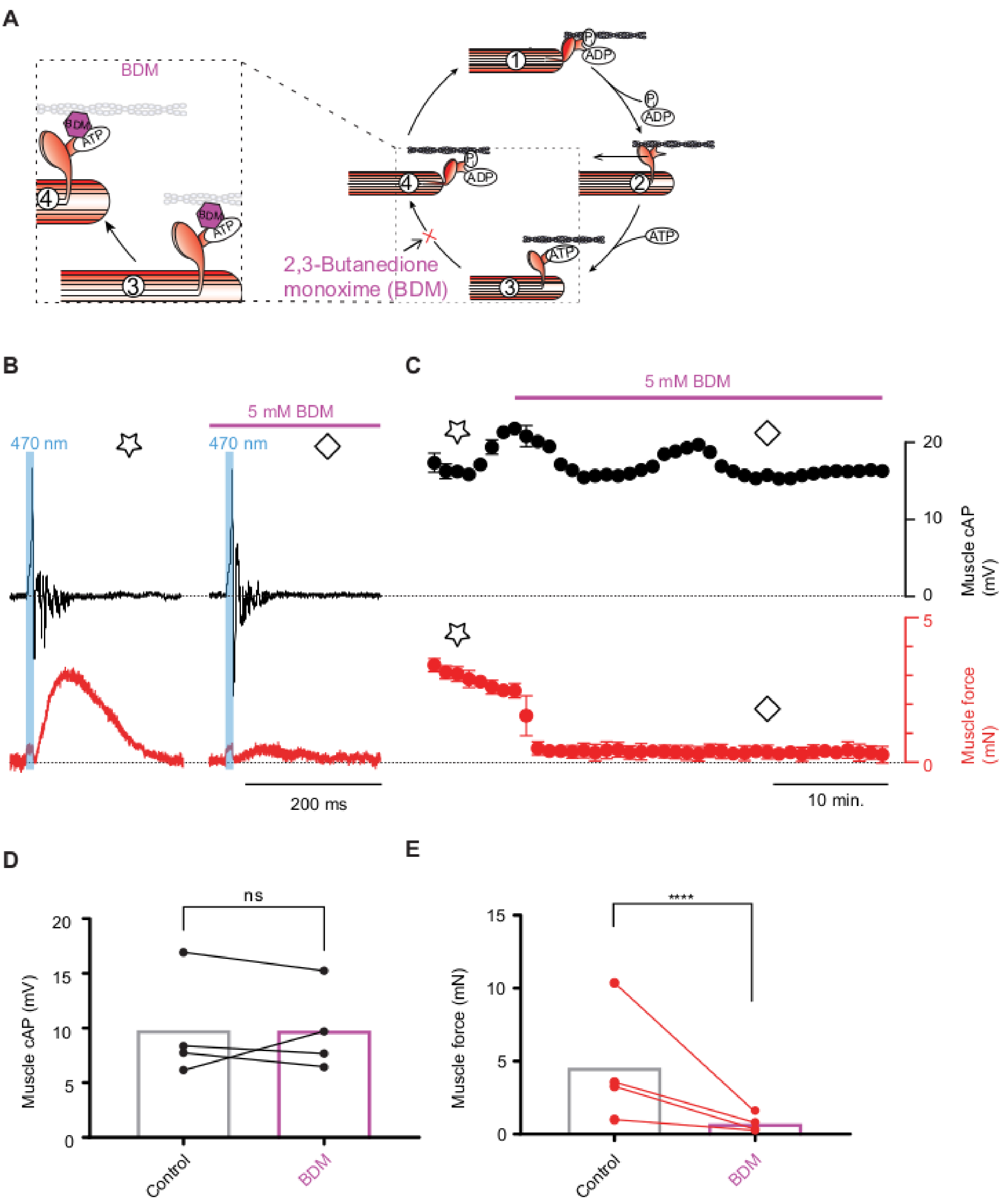

Supplementary Figure 5: a) Principle of muscle excitation-contraction uncoupling. BDM, a Myosin ATPase inhibitor, blocks the phosphorylation of the myosin head (insert), which prevents muscle contraction. b) Representative examples of light-induced muscle excitation in control conditions (left) and in the presence of BDM (right). Black traces: Muscle compound action potential (cAP); red traces: Force generated by the muscle. c) Time course for the changes in cAP and muscle force during the application of BDM (5mM). Each point is an average of 30 consecutive stimulations applied at 0.5 Hz. BDM abolished muscle contraction but had no effect on the cAP. d) Mean and individual amplitudes of the cAP for all muscles tested in control conditions and in the presence of BDM (n=4; no significant difference: p=0.8445, Wilcoxon test). e) Mean and individual amplitudes of muscle force in control condition and in the presence of BDM (n=4; significant decrease: p<0.0001, Wilcoxon test).

4 Movement Inhibition

A challenge for recording action potentials in myocytes is the mechanical artifact produced by the shortening of the cells. The action potential that propagates along the surface of muscle fibers is coupled to the contraction via a complex molecular cascade that involves several proteins. In brief, the impulse invades the depth of myocytes along the transverse tubules and induces the release of Ca^{2+} from intracellular stores. Ca^{2+} binds to troponin, which allows the interaction of actin with the head of myosin. The subsequent movement of myosin head is responsible for the shortening of the muscle. During this power stroke, the myosin heads releases ADP (Supplementary Figure 5,a) allowing for the binding of an ATP molecule. The myosin head has an ATPase activity which induces the dephosphorylation of ATP into ADP. The resultant release of energy moves the head of the myosin molecule back to its original position. We tested if blocking the ATPase activity with 2,3-Butanedione monoxime (BDM) was sufficient to uncouple the excitation to the contraction. We found that few minutes after addition of BDM (5mM) to the extracellular medium, the muscle twitch triggered by blue light was eliminated (control: 4.55 $\pm$3.60 mN, BDM: 0.72$\pm$0.58 mN, n=4, p<0.0001, Wilcoxon test; Supplementary Figure 5, b)-e). By contrast, the compound action potential recorded at the surface of the muscle remained unaffected (control: 9.79 $\pm$3.8 mV, BDM: 9.76 $\pm$3.44 mV, n=4, p=0.42, Wilcoxon test). This was implemented in the experiment in the main paper from Muscle 2 only.

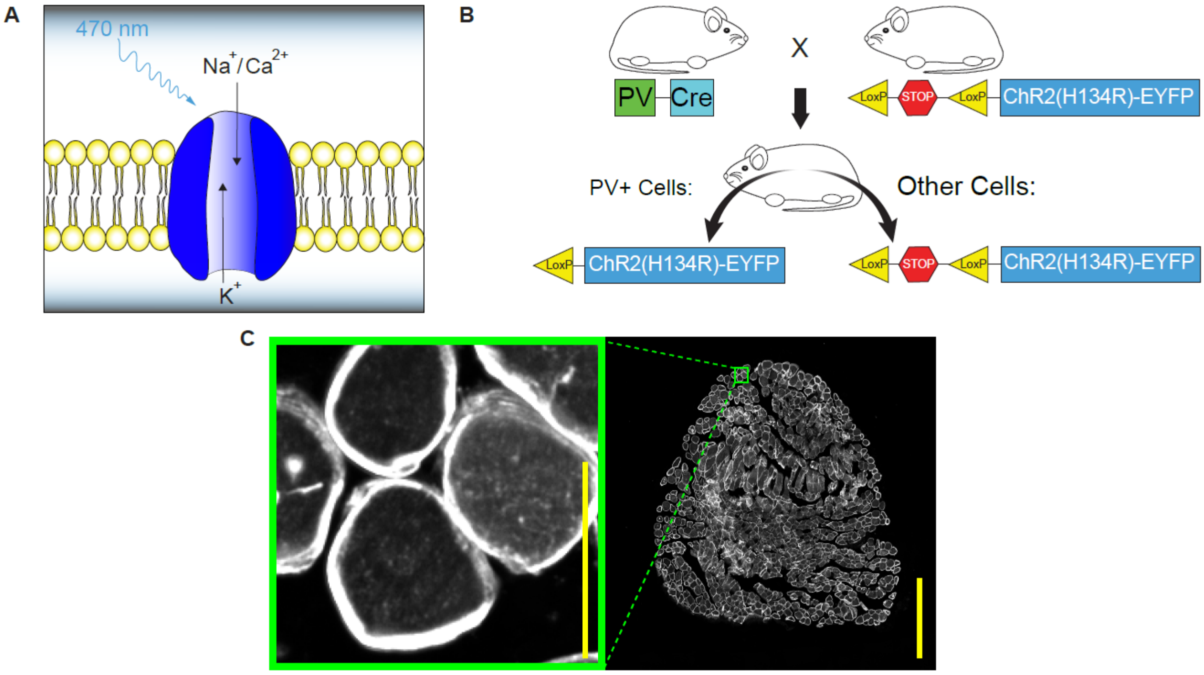

Supplementary Figure 6: Illustration of the basic principle of optogenetics. a) Blue light of 470 nm induces a conformational change of channelrhodopsin allowing cations to flux through the plasma membrane. The depolarization mediated by light-activation is primarily driven by an inward current mediated by Na+ ions. b) Offspring from PV-Cre and ChR2(H134R)-EYFP mice express the light sensitive cation channel channelrhodopsin in parvalbumin positive cells. c) Channerhodpsin2 expression in EDL muscle fibers from PV-cre::ChR2-EYFP mice. Confocal image of a transverse section of EDL muscle (right) and a selection of myocytes showing bright fluorescence from the ChR2/EYFP expression in the sarcolemma (left). Scale bar right: 500μm and right: 50μm.

¤

5 Optogenetics

The muscle was stimulated optogenetically using blue light from a 470nm LED. Experiments were performed on genetically modified mice in which Channelrhodopsin 2 (ChR2), a light-gated cation channel (Supplementary Figure 6,a), was expressed under the control of the parvalbumin (PV) promoter, a calcium binding protein expressed by fast contracting muscle fibers ([1]). We took advantage of the Cre-Lox recombination technology to insert the ChR2 gene in fast-twitch myocytes. This was achieved by crossing Gt(ROSA)26Sortm32(CAG-COP4*H134R/EYFP)Hze mice with Pvalbtm1(cre)Arbr mice (Supplementary Figure 6,b). The offspring expressed both the yellow fluorescent protein (YFP) and ChR2 in their myocytes (Supplementary Figure 6,c).

Immunohistochemistry for Supplementary Figure 6,c): Adult PV-cre::ChR2-EYFP mouse, were euthanized by cervical dislocation. EDL muscles were dissected and fixed in 4% (w/v) paraformaldehyde in phosphate-buffered saline (PBS) for 2 hours, then rinsed in PBS and cryoprotected in 30% (w/v) sucrose in PBS overnight at 4 °C. The EDL muscles were embedded in OCT mounting medium and cut into 20 μm thick transverse sections using a cryostat. Sections were blocked in PBS containing 5% (v/v) fetal bovine serum and 0.2% (w/v) Triton X-100 (blocking solution) and incubated overnight at 4 °C with chicken anti-GFP (Abcam) as primary antibody diluted 1:1000 in blocking solution. After rising with blocking solution the sections were incubated for 2 hours at room temperature with Anti-chicken Alexa 488 (ThermoFisher) as secondary antibody diluted 1:500 in blocking solution. Sections were rinsed in blocking solution, mounted in Vectashield medium and scanned on a confocal microscope (Zeiss) using a 10x objective.

6 Modeling

The modeled magnetic field was calculated as being directly proportional to the temporal derivative of the compound action potential (cAP) of the muscle, and the cAP was calculated by superimposing multiple single fiber AP:s from a muscle model incorporated with a channelrhodopsin mechanism, which fire at different times over the course of the light stimulation.

The action potential (AP) of a single fiber from an EDL muscle in mouse was modeled in NEURON[2] using ion mechanisms and parameters taken from Cannon et al.[3]. The AP was triggered with a current (I_{ChR2}) coming from the opening of ChR2 due to light activation. I_{ChR2} was modeled based on the work by Nikolic et al.[4] who showed how I_{ChR2} behaves over time in a single neuron. In the present study, this was simplified by assuming a two-state model of the amount of open ChR2 in order to reduce the number of parameters that had to be fitted, without significantly altering the dynamics behavior at short illumination times ($<$10 ms). The ChR2-current for short illumination times increases rapidly during the illumination, and more slowly exponentially decays to zero post-illumination. The simplified two-state model,

describing the amount of open (N_{op}) and closed channels (N_{cl}), is defined as:

$$\frac{dN_{opt}}{dt} = K_a N_{cl} - K_d N_{op} \tag{1}$$

Where N_{cl}=1-N_{op}, K_d is the closing rate of the channels, and K_a is the rate of ChR2 activation which is dependent on the absorbed photons (F) and the quantum efficiency of the ion channels (ε), and the opening time of the ChR2-channels τ_{ChR2}. This activation rate was presented in Nikolic et al.[4] as:

$$K_a(t) = \begin{cases} F^* \left(1 - e^{\frac{-(t-t_{on})}{\tau_{ChR2}}}\right), t_{on} < t < t_{off} \text{ LED on} \\ F^* \left(e^{\frac{-(t-t_{off})}{\tau_{ChR2}}} - e^{\frac{-(t-t_{on})}{\tau_{ChR2}}}\right), t \geq t_{off} \text{ LED off} \end{cases} \tag{2}$$

Where F^*=εF. The current contribution to the fiber from the ChR2 is then proportional to the number of open ChR2 channels, thus: I_{ChR2}=$I_{max}N_{op}$, where I_{max} was set fixed at 1nA. The resulting membrane potential of this fiber can be seen in Supplementary Figure 7.

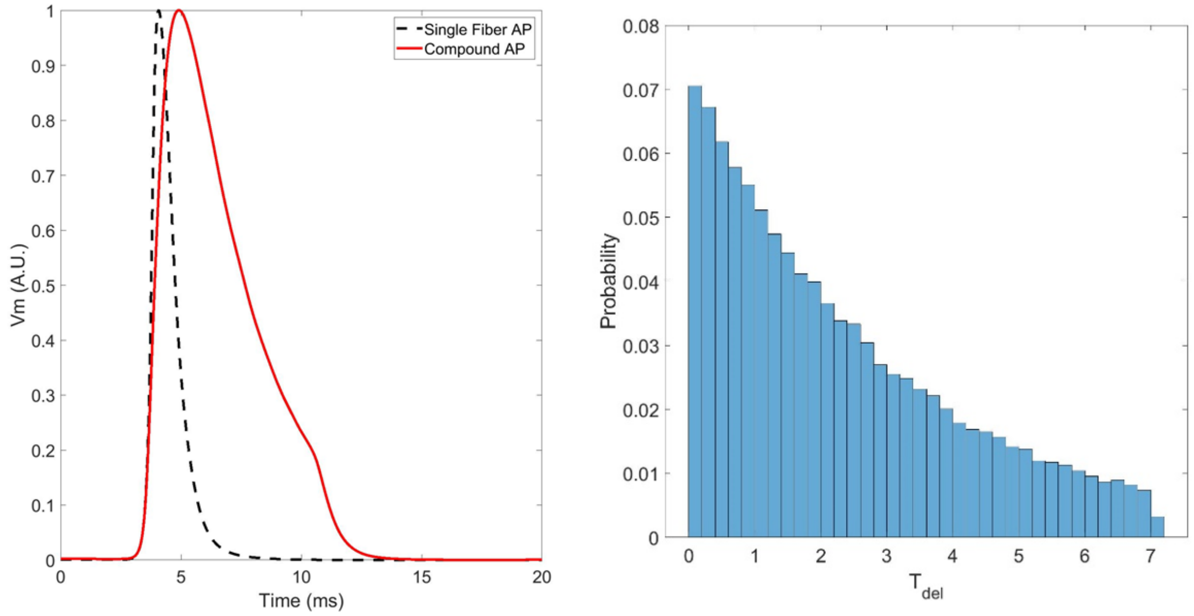

Supplementary Figure 7: Left: Model of an EDL muscle membrane potential of a single muscle fiber triggered optogenetically, and the compound AP of all stimulated muscle fibers. Right:Probability distribution of starting time over the time the LED is on with 1000 trials.

Since the probability of the opening of a ChR2 protein is dependent on the intensity of the light[4], it is expected to find a higher number of fibers firing in regions where the light intensity is higher, i.e. on the surface of the muscle. The light intensity decay exponentially with penetration depth[5], and thus the probability that an AP fires decreases with penetration depth as well. Furthermore, as can be shown from solving the single fiber AP equations presented above, a decreased light intensity ($\propto F^*$) increases the time it takes from illumination to triggering of the AP (T_{del}). Thus, both the number of APs fired at a specific depth and the time delay decreases

exponentially with penetration depth, which means that we can assume that the distribution of APs fired at a specific time can also be described by a exponentially decaying probability function, such that P(T_{del}=t)=$\lambda e^{(-\lambda t)}$, where the parameters λ is related to penetration depth of the light. However, an AP can only fire given that the light intensity is sufficiently strong during the 5ms illumination time to elicit an AP, and therefore it must exist some minimum F^* which corresponds to a maximum delay time ($T_{del,max}$), at which point the probability of stimulating an AP goes to zero. Thus:

$$P(T_{del} = t) = \begin{cases} \lambda e^{-\lambda t}, & t < T_{del,max} \\ 0, & t \geq T_{del,max} \end{cases} \tag{3}$$

The cAP (Supplementary Figure 7 , left) is then calculated as the sum of all N spikes fired at different times according to this time distribution.The maximum delay time means that there is a sudden drop in the cAP at $T_{del,max}$ (as seen in SI Figure 7). This sudden drop in the cAP translates into an apparent dip in the modeled magnetic field data when the signal returns to baseline value (as seen in the model fit in the main text).

Magnetic field calculations: The magnetic field from the muscle is calculated as per Barry et al. [6], i.e. as the derivative of the potential:

$$B_{sim}(t) = s\frac{dV_{tot}}{dt} \tag{4}$$

where V_{tot} is the cAP of the signal (Supplementary Figure 7, left), s is a scaling factor which depends on the geometry of the experiment (muscle size, exact muscle to sensor distance, etc.), the number of fibers firing, and screening effects. Since many of these parameters were unknown, this factor was simply set such that the peak-to-peak amplitude of the simulated magnetic field (B_{sim}) was equal to that of the measured magnetic field.

Although the majority of the model parameters presented here were taken from literature values (i.e. EDL muscle Hodgkin-Huxley parameters[3], and certain ChR2 model parameters [4]), some parameters were unknown and thus had to be optimized by fitting the modeled data to the measured magnetic field. The fitted parameters are: the number of AP:s being fired (N), the maximum time delay ($T_{del,max}$) and the rate parameters of the time distribution λ. N was restrained between 1 and 1000 (approximately the maximum number of fibers in a muscle), $T_{del,max}$ was constrained within the shortest and longest delay obtained by changing the F^* parameter in the single fiber AP model (approx. 0.5 and 9 ms respectively), and λ was unconstrained. The parameters used for the data in the main paper were N=74, λ=2.9ms^{-1} and $T_{del,max}$=7.1ms. The fits are presented overlaid on the experimental data in the main paper.

The presented model was made to qualitatively fit the magnetic field from an optogenetically stimulated muscle, based on a few assumptions regarding the system. Single fiber AP models, and ChR2 dynamics are well described in the literature [3, 4] and the magnetic field calculations were based on simple scaled time derivatives [6] of the compound AP. The time distribution of

the AP firing was based on some simple assumptions about the stochastic nature of optogenetic stimulation and about the distribution of light within a muscle. The fitted model parameters were restrained such that they would not deviate significantly from reasonable assumptions, or previously reported values, and ultimately allowed a reasonable qualitative model of the measured magnetic field.

References

[1] M. R. Celio and C. W. Heizmann, Nature **297**, 504 (1982).

[2] N. T. Carnevale and M. L. Hines, *The NEURON Book* (Cambridge University Press, 2006).

[3] S. Cannon, R. Brown, and D. Corey, Biophysical Journal **65**, 270 (1993).

[4] K. Nikolic, N. Grossman, M. S. Grubb, J. Burrone, C. Toumazou, and P. Degenaar, Photochemistry and Photobiology **85**, 400 (2009).

[5] O. Yizhar, L. E. Fenno, T. J. Davidson, M. Mogri, and K. Deisseroth, Neuron **71**, 9 (2011).

[6] J. F. Barry, M. J. Turner, J. M. Schloss, D. R. Glenn, Y. Song, M. D. Lukin, H. Park, and R. L. Walsworth, Proceedings of the National Academy of Sciences **113**, 14133 (2016).
